# Potential of MALDI-TOF MS as an alternative approach for capsular typing *Streptococcus pneumoniae* isolates

**DOI:** 10.1038/srep45572

**Published:** 2017-03-28

**Authors:** Tatiana C. A. Pinto, Natalia S. Costa, Luciana F. S. Castro, Rachel L. Ribeiro, Ana Caroline N. Botelho, Felipe P. G. Neves, Jose Mauro Peralta, Lucia M. Teixeira

**Affiliations:** 1 Instituto de Microbiologia Paulo de Goes, Universidade Federal do Rio de Janeiro, Rio de Janeiro, RJ, Brazil; 2 Universidade Federal Fluminense, Niterói, RJ, Brazil

## Abstract

*Streptococcus pneumoniae* can be classified in more than 90 capsular types, as traditionally determined by serological methods and more recently by PCR-based techniques. Such methods, however, can be expensive, laborious or unable to accurately discriminate among certain serotypes. Therefore, determination of capsular types, although extremely important for epidemiological purposes and for estimating the impact of pneumococcal conjugate vaccines, is mainly restricted to research laboratories, being rarely performed in the clinical setting. In the present study, MALDI-TOF MS was evaluated as an alternative tool to characterize 416 pneumococcal isolates belonging to serotypes 6A, 6B, 6C, 9N, 9V or 14. For MALDI-TOF MS analysis, each isolate was submitted to an extraction protocol using formic acid and acetonitrile. Measurements were performed with a Bruker Microflex LT mass spectrometer using default parameters and generating spectra in the range of 2,000–20,000 *m/z*. Spectra were analyzed with the BioNumerics software v7.6. Isolates were mainly distributed according to the capsular type in a Neighbor Joining tree and serotypes investigated were successfully discriminated by the presence/absence of 14 selected biomarkers. The results suggest that MALDI-TOF MS is a promising alternative for typing pneumococcal strains, highlighting its usefulness for rapid and cost-effective routine application in clinical laboratories.

*Streptococcus pneumoniae* is a major human pathogen that can be also found as nasopharyngeal colonizers among variable numbers of asymptomatic individuals worldwide^1^. The polysaccharide capsule is the basis for characterizing pneumococcal isolates into serotypes, and represents the target of pneumococcal conjugate vaccines (PCV) currently available^2^. Although more than 90 capsular types have been reported, a restricted number of serotypes stands out for their high prevalence in both pneumococcal disease and nasopharyngeal colonization, as well as for association with antimicrobial resistance. Serotype 14 and serotypes comprised by serogroups 6 and 9 are important examples, and most of them have already been included in PCVs^2^,^3^,^4^. In Brazil, the first PCV introduced in the National Immunization Program for routine free of charge childhood vaccination was the 10-valent vaccine (PCV10) in 2010, comprising serotypes 1, 4, 5, 6B, 7F, 9V, 14, 18C, 19F, and 23F^5^. Simultaneously, the 13-valent vaccine (PCV13), containing the additional serotypes 3, 6A, and 19A, was made commercially available in private clinics.

Surveillance of pneumococcal serotypes is important, among other purposes, to evaluate vaccination effects. The standard methodology for capsular typing is the Quellung reaction, which is mostly limited to reference centers due to the strict requirements regarding preparation and quality control of antisera and training of the staff involved. Latex agglutination, PCR amplification or sequencing of capsular genes represent some of the alternative approaches^2^. These methods, however, can be expensive and laborious; and some of them do not accurately discriminate among serotypes within a same serogroup, such as serogroup 6. Hence, determination of capsular types can be restricted, especially in laboratories of resource-limited countries, being rarely performed in the clinical setting.

MALDI-TOF MS is an emerging technology that has been increasingly used in clinical laboratories for rapid bacterial identification^6^. Moreover, it has also showed the potential for typing applications among streptococcal species^7^,^8^,^9^,^10^. In the present study, MALDI-TOF MS was evaluated as an alternative tool to characterize *Streptococcus pneumoniae* isolates according to the capsular type.

## Methods

A total of 416 *Streptococcus pneumoniae* isolates were analyzed, including 141 strains of serotype 14, 49 strains of serotype 6A, 143 strains of serotype 6B, 38 strains of serotype 6C, 24 strains of serotype 9N and 21 strains of serotype 9V.

All the isolates were recovered during surveillance studies performed by our group or were received from various national health institutions for species confirmation and capsular typing, between 1988 and 2011. Isolates obtained from diseased individuals, including children and adults, were recovered from clinical specimens taken as part of standard patient care procedures and did not require ethical approval for their use. Isolates from carriage studies, including both children and adults, were recovered from clinical specimens as approved by the ethics committees of the institutions involved. The isolates were previously identified by phenotypic tests, including observation of colony morphology and type of hemolysis on blood agar plates; cellular characteristics after Gram stain; optochin susceptibility and bile-solubility^11^. The capsular types were previously determined for all 416 strains by the Quellung reaction with antisera kindly provided by the *Streptococcus* Laboratory at the Centers for Disease Control and Prevention (CDC, GA, USA), and also by multiplex PCR for a fraction of the isolates^12^,^13^,^14^,^15^,^16^,^17^.

For MALDI-TOF MS analysis, a quick extraction protocol was performed for all isolates as follows. After overnight growth, five bacterial colonies were suspended in 5 μl of formic acid 70% (Tedia, Cincinatti, OH, USA), which was then vigorously homogenized during 10 s. Subsequently, 5 μl of acetonitrile (Tedia) were added to the suspension, which was then gently homogenized. The suspension was centrifuged at 5000 rpm for 3 min, and 1 μl of the supernatant was poured in a spot of the target plate (MSP 96 target polished steel BC, Bruker Daltonics, Germany). After drying, each spot was covered with 1 μl of CHCA matrix (α-Cyano-4-hydroxycinnamic acid; Bruker Daltonics).

Measurements were performed with the Microflex LT mass spectrometer (Bruker Daltonics) and Biotyper software using the default parameters (laser frequency of 60 Hz, ion source voltages of 2.0 and 1.8 kV, and lens voltage of 6 kV), which generated spectra in the range of 2,000–20,000 *m/z*. Spectra were then exported to the BioNumerics software v7.6 (Applied Maths, Ghent, Belgium), where the raw spectra were preprocessed and normalized using default parameters (baseline subtraction was performed with Running Rolling disc, and peak detection was performed using a signal to noise ratio of 10). After preprocessing, spectra were submitted to peak matching using a tolerance of ±0,002 *m/z*.

All biomarkers automatically detected by BioNumerics were exported to a spreadsheet containing all strains (see Supplementary Spreadsheet S1). This spreadsheet was visually analyzed by counting the number and calculating the percentage of strains containing a certain biomarker within each serotype. After analysis, a set of biomarkers was selected to constitute serotype-specific profiles.

In addition, a Neighbor Joining tree based on Pearson coefficient was constructed using BioNumerics.

Diversity of serotypes and MALDI profiles was assessed by the Simpson’s Index of Diversity (SID)^18^, and congruence between serotype and MALDI profile was estimated by the Adjusted Wallace Coefficient (AW), with 95% confidence intervals (95% CI)^19^. SID and AW were calculated using the online tool available at http://www.comparingpartitions.info.

Reference or internal control *S. pneumoniae* strains (strain ATCC700902 of serotype 14, strain Sp1019 of serotype 9N, strain ATCC700671 of serotype 9V, strain ATCCBAA659 of serotype 6A, strain ATCC700675 of serotype 6B and strain 2008008385 of serotype 6C) were also analyzed.

## Results and Discussion

**Figure 1:**
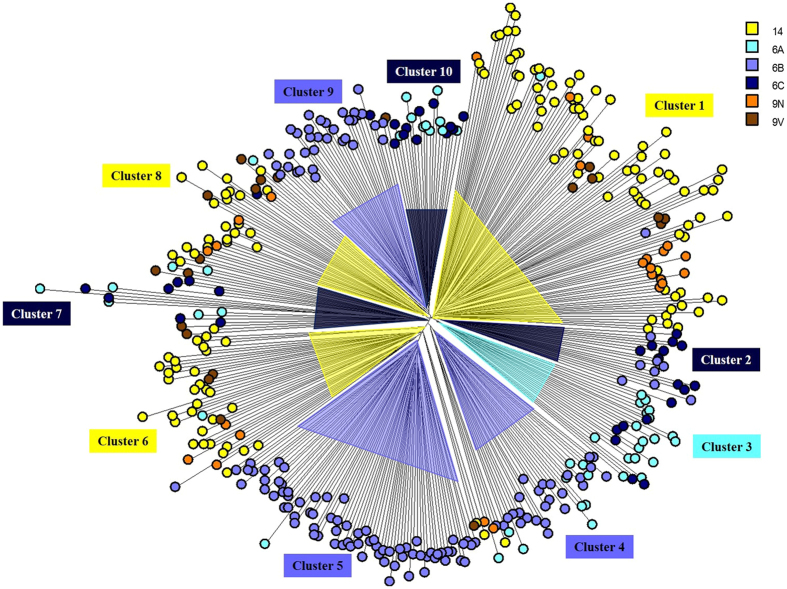
Neighbor Joining tree based on Pearson correlation constructed with MALDI-TOF MS spectra of 416 pneumococcal isolates showing the 10 major clusters and distribution according to the capsular type. Each node represents the spectrum of a pneumococcal strain, and different serotypes (as determined by the Quellung reaction) are shown in different colors (see legend).

In the Neighbor Joining tree, isolates were distributed mainly according to the capsular type (Fig. 1), and 12 clusters were detected, including 10 major and two minor clusters. Distribution of isolates according to serotype and major MALDI clusters is shown in Table 1.

Isolates belonging to certain capsular types, such as 9N and 9V, showed a more random distribution across the tree, while strains belonging to other serotypes, such as 6B and 14, showed a tendency to a more homogeneous clustering. Interestingly, capsular types 9N and 9V were the serotypes less represented in the study, comprising around 20 isolates each. In turn, serotypes 14 and 6B included the largest numbers of strains evaluated in the present study (around 140 each), suggesting that clustering might be enhanced as more strains are included in the analysis.

Congruence between MALDI-TOF cluster and capsular type was observed (AW = 0.604; 95% CI 0.527–0.681). In addition, a higher diversity (SID = 0.863) was observed regarding MALDI profiles when compared to serotype distribution (SID = 0.742). These observations indicate that MALDI-TOF MS results are congruent with capsular typing, and are also more discriminatory, suggesting that this technique might be useful to predict intra-serotype variations, such as those related to distribution of clones and antimicrobial susceptibility profiles.

In general, isolates belonging to serotypes 9N and 9V were mainly clustered together with serotype 14 isolates. Likewise, serotype 6A strains were mostly allocated together with serotype 6C isolates. These results corroborate the close genetic relationship between serotype 14 and serogroup 9^20^, as well as between serotypes 6A and 6C^21^,^22^, suggesting that MALDI-TOF MS also has potential for inferring phylogenetic relationship.

**Figure 2:**
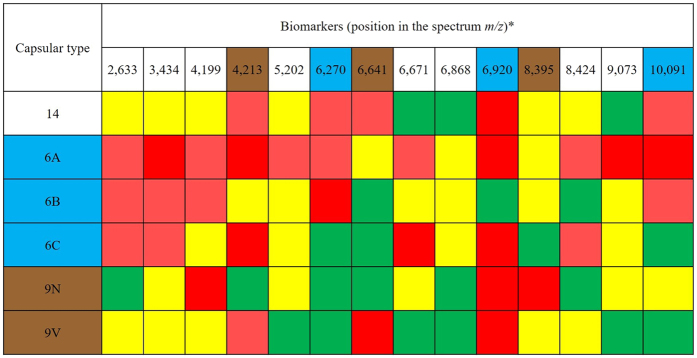
Profile of 14 MALDI-TOF MS biomarkers selected to differentiate pneumococcal capsular types. *Position in the spectra using a tolerance of ± 0,002 *m/z*. Green indicates that the biomarker is present in more than 90% of the strains of a given serotype; Yellow, present in 70–89% of the strains; Light red, present in 50–69% of the strains; Red, absent in all strains. The sets of 3 biomarkers useful to discriminate among serotypes within serogroups 6 and 9 are highlighted in blue and brown, respectively.

A total of 35 peaks (or biomarkers) were detected, and they ranged from 2,633 to 10,405 *m/z* (see Supplementary Spreadsheet S1). After analysis of these biomarkers, 14 were selected to compose serotype-specific profiles (Fig. 2). The profiles (presence/absence) of selected biomarkers were successful for discriminating all serotypes evaluated, even those that presented a more scattered distribution in the Neighbor Joining tree, such as 9N and 9V (Fig. 1).

Selected biomarkers were also classified according to their distribution within each serotype, as follows: green when present in more than 90% of the strains of a given serotype; yellow when present in 70–89% of the strains; light red when present in 50–69% of the strains; and red when absent in all strains of a given serotype. To perform a preliminary validation, these biomarkers were investigated in reference or internal control *S. pneumoniae* strains of each serotype evaluated and all of them were shown to possess the expected profile.

Additionally, differentiation among serotypes within a single serogroup (as in serogroups 6 and 9) was achieved by the analysis of sets of 3 biomarkers (Fig. 2). Serogroup 9 comprises serotypes 9A, 9L, 9N, and 9V; likewise, serogroup 6 includes serotype 6A, 6B, 6C, and 6D^2^. Serotypes 9A, 9L, and 6D are still rarely detected^13^,^15^. In our collection, only three strains belonged to serotype 9A, two to serotype 9L and only one to serotype 6D. Therefore, due to the lack of representativeness, strains belonging to such serotypes were not included in the main analysis. However, the profile of the 3-biomarker sets used to differentiate serotypes within serogroups 6 and 9 was also evaluated in those strains and they indeed exhibited different profiles, being distinguished from the other serotypes in the same serogroup. Although a higher number of isolates belonging to those less commonly reported serotypes should be evaluated, our results suggest that MALDI-TOF MS could work as a complementary methodology to determine serotype once serogroup has been established by other means, such as PCR.

Determination of pneumococcal capsular types is essential for different purposes. Specifically, discrimination among serotypes comprised by the same serogroup is crucial since some of them are included in PCVs (such as serotypes 6A and 6B of serogroup 6 and serotype 9V of serogroup 9). On the other hand, serotypes 6C and 9N are not present in any of the available conjugate vaccines and, thus, have the potential to emerge in this scenario^2^. Indeed, the emergence of serotype 6C has been detected in the USA after PCV7 implementation^14^,^15^.

Therefore, a more suitable and accessible methodology to determine the capsular type of pneumococcal isolates is needed. Recently, MALDI-TOF MS was evaluated for this purpose, showing the potential for differentiating ten major pneumococcal serotypes in Japan, including serotypes 3, 6B, 15A, 15C, 19A, 19F, 23A, 24F, 35B, and 38^7^. However, the ability to distinguish the various serotypes comprised by serogroup 6, as well as to differentiate serotype 14 and those within serogroup 9 investigated in the present study, has not been previously evaluated.

This work contributes for the development and evaluation of alternative methodologies for the capsular typing of pneumococcal strains, indicating that MALDI-TOF MS is a promising tool. Nevertheless, as every novel and recently proposed scientific approach, it needs external validation in order to assess the reproducibility of results among pneumococcal strains recovered from other places of the world. Still, the observation that a quick and simplified extraction protocol was sufficient to generate high quality MALDI-TOF spectra highlights its usefulness for rapid and cost-effective routine application in clinical and resource-limited laboratories.

## Additional Information

**How to cite this article**: Pinto, T. C. A. *et al*. Potential of MALDI-TOF MS as an alternative approach for capsular typing *Streptococcus pneumoniae* isolates. *Sci. Rep.* **7**, 45572; doi: 10.1038/srep45572 (2017).

**Publisher's note:** Springer Nature remains neutral with regard to jurisdictional claims in published maps and institutional affiliations.

## Supplementary Material

Supplementary Dataset S1

## Figures and Tables

**Table 1:** Distribution of *Streptococcus pneumoniae* isolates belonging to each capsular type investigated in the present study, according to the MALDI-TOF MS cluster.

Serotype	N	Percentage (number) of strains belonging to each major MALDI cluster										SID^a^
		Cluster 1	Cluster 2	Cluster 3	Cluster 4	Cluster 5	Cluster 6	Cluster 7	Cluster 8	Cluster 9	Cluster 10	
14	141	61% (86)	None	None	None	None	22.7% (32)	0.7% (1)	13.5% (19)	None	None	0.562
9N	24	62.5% (15)	None	None	None	None	16.7% (4)	None	16.7% (4)	None	None	0.607
9V	21	28.6% (6)	None	None	None	None	14.3% (3)	23.8% (5)	23.8% (5)	None	4.7% (1)	0.819
6A	49	2% (1)	None	36.7% (18)	18.4% (9)	2% (1)	2% (1)	14.3% (7)	2% (1)	None	20.4% (10)	0.783
6B	143	0.7% (1)	5.6% (8)	None	17.5% (25)	51% (73)	0.7% (1)	None	None	23.8% (34)	None	0.654
6C	38	None	28.9% (11)	15.8% (6)	None	None	None	21% (8)	2.6% (1)	2.6% (1)	28.9% (11)	0.782
Total	416	109	19	24	34	74	41	21	30	35	22	—

^a^Simpson’s Index of Diversity.
